# “Less Than A Wife”: A Study of Polycystic Ovary Syndrome Content in Teen and Women’s Digital Magazines

**DOI:** 10.2196/jmir.5417

**Published:** 2016-06-02

**Authors:** Ninive Sanchez, Hillary Jones

**Affiliations:** ^1^ School of Social Work University of Missouri Columbia, MO United States; ^2^ Catholic Community Services of Western Washington Newcastle, WA United States

**Keywords:** polycystic ovary syndrome, digital magazines, women's health, edutainment

## Abstract

**Background:**

Polycystic ovary syndrome (PCOS) is a major public health problem that affects women’s physical and mental health. According to the US National Institutes of Health Office of Disease Prevention, there is a need to improve public awareness of the syndrome among health care providers and the public. Women’s magazines are a type of “edutainment” that publish health content in addition to beauty, fashion, and entertainment content. These media have the potential to expose primarily female readers to content on PCOS and influence readers’ beliefs and attitudes about women with PCOS.

**Objective:**

The objective of this study was to explore how digital (online) teen and women’s magazines portray women with PCOS.

**Methods:**

We used data from the Alliance for Audited Media to identify popular digital teen and women’s magazines with circulation rates ≥1,000,001. We also included magazines with circulation rates 100,001–1,000,000 directed toward racial and ethnic minority readers. A search of magazine websites over a 1-month period in 2015 yielded 21 magazines (eg, Glamour, Cosmopolitan en Español, Essence, and O, The Oprah Magazine) and 170 articles containing “PCOS” and “polycystic ovary syndrome.” Textual analysis using a grounded theory approach was used to identify themes.

**Results:**

Articles depicted PCOS symptoms as a hindrance to women’s social roles as wives and mothers and largely placed personal responsibility on women to improve their health. To a lesser extent, women were depicted as using their personal experience with PCOS to advocate for women’s health. Experiences of Latina and African American women and adolescents with PCOS were absent from women’s magazine articles.

**Conclusions:**

The findings can inform health education programs that teach women to be critical consumers of PCOS-related content in digital women’s magazines. Future research on PCOS content in digital teen and women’s magazines can help researchers, patients, and consumer groups engage with the media to increase public awareness of PCOS.

## Introduction

In the United States, 35% of adults, or about 1 in 3 Americans, have used the Internet to find out what medical condition they or someone else might have [1]. Younger age groups and women increasingly use the Internet and social media to access health-related information [2,3]. Among the people searching for health-related information online are women typing “PCOS,” “polycystic ovaries syndrome,” and “PCOS symptoms” [4].

### Overview of PCOS

Polycystic ovary syndrome (PCOS) is a complex, chronic condition the US National Institutes of Health (NIH) Office of Disease Prevention has described as a major public health problem for women in the United States. PCOS is characterized by a range of symptoms including irregular or no menstrual periods, excess hair growth on the face and body (hirsutism), weight gain, acne, ovarian cysts, and alopecia [5]. PCOS can also increase women’s risk of type 2 diabetes mellitus [6], cardiovascular disease [7], infertility [8], anxiety and depression [9-12], and poor health-related quality of life [13].

PCOS can begin in adolescence [14] and worsen across the life course if untreated or poorly managed [15,16]. The prevalence of PCOS among adult women (18-45 years of age) in the United States is about 7% [17]. However, research with a community sample of women (27-34 years of age) in the United Kingdom suggests that the prevalence of PCOS may be even higher, from 7.8% to 20.6%, depending on which of 3 diagnostic criteria for PCOS is used. Additionally, using 2 of these criteria, NIH and Rotterdam, about 70% of women with PCOS symptoms are undiagnosed [18]. See Goodman et al [19] and Rosenfield [20] for a review of the diagnosis and treatment of PCOS in adolescents and adults.

The 2012 NIH Evidence-based Methodology Workshop on Polycystic Ovary Syndrome recommended establishing “multidisciplinary programs to improve public and health care provider awareness and management for women who currently have the syndrome” [5]. The lack of awareness about PCOS is partly due to the condition’s misleading name, which suggests that it is a problem of the ovaries; however, the presence of polycystic ovaries alone does not indicate that a woman has PCOS [5]. Additionally, although PCOS is a highly prevalent condition, it does not have the “celebrity” status of other well-known conditions [21].

### PCOS Online

Women with PCOS describe the process of searching for answers to their symptoms as emotional, confusing, frustrating, devastating [22], and exhausting [23]. In addition to seeing several doctors before receiving a diagnosis, women report diagnosing themselves by searching for information online [24]. Women may search the Internet for information on PCOS they cannot find or understand in books or other print sources [25], and when they do not receive sufficient information and support from health care providers [23]. In addition, the Internet provides women with a convenient, private, and accessible way to access information on symptoms such as obesity and hirsutism (excessive hair) that may be too embarrassing to discuss in person [25]. Women communicate and share experiences with PCOS via chat groups, email lists [25], online support groups [23], and social networking sites like Facebook and Twitter [4].

Research on online information about PCOS has primarily focused on accuracy of this coverage. Information on PCOS appears on social networking sites, government health and professional associations’ websites, and nonprofit organizations’ websites [4,26], and websites vary regarding PCOS symptoms, long-term health effects, and management strategies [4,27]. Websites also tend to omit information on authors, editorial review process, publication date, strength of evidence reported (eg, use of randomized controlled trials), PCOS treatment based on practice guidelines [27], privacy and confidentiality of personal data, and sources [4].

### Media and Health

Media do not just provide consumers with health information, however; they also create social constructions of health, illness, and medical care that have implications for how consumers view and manage their health [28]. Teen and women’s magazines have traditionally conflated health and beauty [29,30]. For instance, an analysis of body-related content published from 1993 to 2003 in *Seventeen*, a magazine for adolescent girls, found that content created constructions of desirable bodies as lean, toned, and free of hair and acne [31]. Equating health with beauty can be problematic for readers with PCOS. For instance, teen magazines publish techniques such as hair removal strategies and quick-fix diet and exercise routines readers can follow to achieve the desirable body [31]. Hair removal techniques that do not address the underlying causes of excessive hair growth can mask the severity of PCOS-related hirsutism [32]. Diet and exercise routines to achieve the desirable body in a short amount of time can overlook the importance of long-term lifestyle changes in diet and physical activity needed to manage PCOS. On the other hand, content in women’s magazines that encourages consumers to collaborate with health care providers, be cautious about treatments and medications, and look to friends and family for social support may help consumers manage their health [33].

### Edutainment

Magazines are a type of entertainment-education or “edutainment,” which refers to the placement of educational content in entertainment messages [34]. Magazines publish health content in addition to beauty, fashion, and entertainment content. Teen magazines have published health content on topics such as sexually transmitted infections, gynecological visits, and birth control [35], and women’s magazines have published on breast cancer [36], heart disease, osteoporosis, and depression [33]. Interestingly, there is evidence in the literature that magazine readers come across content on PCOS. As an adolescent pointed out,

So I’m sitting inside the waiting room and I’m reading through and looking through magazines, and I came across an article in the Seventeen magazine: polycystic ovary disease. And a light bulb went off in my head. I’m like, hey, I gained weight for no reason. I have hair all over my face and it’s popping up in places I don’t want the hair to pop up. And so, I took it to my gynecologist and she was like, “That’s what you have” [22].

Research also suggests that women with PCOS relate information they see in the media to their health, as the following quote illustrates: “If you look at how the media portrays what is feminine, lack of body hair, thinness, and a beautiful complexion, you don’t have these with PCOS and wish you did” [22].

While several health topics have been featured across a range of edutainment [34], researchers have not examined content on PCOS in teen and women’s magazines. These media have the potential to expose primarily female readers to content on PCOS and influence readers’ beliefs and attitudes about women with PCOS. In particular, online magazines, also referred to as digital magazines, are capable of reaching large audiences due to the lower cost of production and distribution, compared with print magazines [37].

This study aimed to understand content on PCOS in digital teen and women’s magazines. The following research questions guided this study: (1) Do digital teen and women’s magazines publish articles on PCOS? (2) Which magazines publish articles on PCOS? (3) How do digital teen and women’s magazines portray adolescents and women with PCOS? (4) What is the discourse and ideology related to health, illness, and gender in these media?

This research can provide insight into health communication on PCOS in digital magazines to which readers are exposed. The findings can inform health education programs that teach women to be critical consumers of PCOS-related content in digital women’s magazines.

## Methods

### Magazine Selection

We used data from the Alliance for Audited Media (AAM) to identify the highest-circulating teen and women’s magazines with digital editions published in the United States. The AAM is a not-for-profit organization founded in 1914 (formerly known as the Audit Bureau of Circulation) that provides audited circulation figures, among other services and data, for newspapers, magazines, and digital media companies in the United States and Canada [38]. Advertising firms use these services and data to obtain details on various media types (eg, magazines, newspapers) to most effectively reach target markets or audiences. AAM is also available to academic institutions with electronic subscriptions to AAM [39]. Researchers, for instance, have used AAM to study the portrayal of gun violence and serious mental illness in news media coverage [40].

Consumer magazines with membership to AAM file publishers’ statements with AAM every 6 months. Publishers’ statements are claims made by consumer magazines that contain information on the publication’s field served and average circulation of print and digital issues. AAM defines field served as the publisher’s description of the markets or occupations whose interest the editorial content is directed toward. In other words, field served describes the publication’s target audience or readership. Additionally, AAM defines digital edition circulation as the distribution of a magazine’s content via electronic means. The digital edition maintains the same identity (eg, the same name and logo characteristics) as the host (or print) publication [38].

First, we identified magazines for this study by selecting AAM’s circulation range of ≥1,000,001. This range was used as an indicator of popular consumer magazines. Second, we retrieved publishers’ statements for magazines that met the circulation criteria. Magazines were selected for inclusion in this study if the publisher’s statement described the field served as women or female teens. This yielded a total of 24 magazines. We also selected magazines with circulation rates 100,001–1,000,000 in an effort to include magazines catering to racial and ethnic minority readers that may not have had the highest circulation rates. Publishers’ statements were also retrieved for these magazines, yielding 3 additional magazines whose target audience or readership was women or female teens.

### Article Selection

We independently searched each of the magazine websites from January 22 to February 25, 2015, to identify articles containing the keywords “PCOS” and “polycystic ovary syndrome.” We selected a 1-month search period to determine whether any content on PCOS was published in teen and women’s digital magazines. We compared the websites and number of articles containing the keywords. This yielded a total of 21 magazines and a total of 170 articles containing the keywords. The magazines were *Better Homes and Gardens*, *Cosmopolitan*, *Cosmopolitan en Español*, *Essence*, *Family Circle*, *Fitness*, *Glamour*, *Good Housekeeping*, *Health*, *MORE*, *O, The Oprah Magazine*, *Parenting*, *Parents*, *Prevention*, *Redbook*, *SELF*, *Shape*, *Teen Vogue*, *Vanidades*, *Woman’s Day*, and *Women’s Health*; 2 of these magazines, *Essence* and *Teen Vogue*, did not contain the keywords in articles, but rather in the reader comments.

### Article Coding and Textual Analysis

We imported the final 170 articles into NVivo 10 for Windows (QSR International) using the NCapture for NVivo add-on in Chrome (Google). NCapture is a Web browser extension that captures Web content (eg, screenshots of webpages) that can then be imported into NVivo 10 for Windows as PDF sources [41]. In 1 instance, there was a video embedded in a Redbook article that had no text in the article except for the article’s title and caption. The video was a total of 2:51 minutes in length and was streamed directly on Redbook’s site, rather than taking the reader to a video-sharing website such as YouTube (YouTube, LLC). We transcribed the video and included the content in the analysis.

The 2 researchers independently coded each of the 170 articles to identify the following 5 primary content areas published in women’s magazines: medical advice, letters or advice, personal stories, advertisements, and visual imagery. Medical advice pieces discussed PCOS in terms of warning signs, treatment, and prognosis. Letters or advice columns were those where questions about PCOS were asked and answered. Personal stories or narratives were based on women’s own experiences with PCOS. Advertisements included health products and services related to PCOS. Visual imagery included images associated with the articles. We focused on the articles containing medical advice, letters or advice, and personal stories or narratives.

The 2 independent coders, both women, conducted a textual analysis using a grounded theory approach to coding to identify themes. The lead researcher identified as Mexican American and had background in psychology, social work, and public health. The graduate student research assistant, who was unfamiliar with the study aims, identified as white and had a background in social work and public health. The lead researcher was fluent in Spanish and the research assistant was proficient.

During the data collection process, each researcher kept memos in which she documented the date the magazine article was retrieved and the titles of the magazine and article. Reflective information such as thoughts and questions that arose during this process were also noted.

Articles were read repeatedly and inductively, and new themes were noted as they emerged. The units of analysis were the article, title, caption, and user comments. Each researcher independently conducted an initial, unrestricted coding of the text in magazine articles by reading the texts line by line. Then, each researcher developed a coding scheme based on her interpretation of the text and memos. The researchers read the magazine articles as cultural artifacts: as texts that provide insight into social practices, representations, and assumptions about society [42].

We held peer debriefings to compare coding, understand each other’s interpretations of the data, integrate categories, and refine the codebook. The codebook contained the thematic categories, examples of text for each category, and codes associated with each category. During these meetings, we viewed the screenshots of the magazine articles and reviewed the memos to contextualize their interpretations. This approach follows Boellstorff and colleagues’ recommendation that data collected in virtual spaces (eg, screenshots and chat logs) be used in conjunction with field notes, not as a substitute for field notes [43].

Finally, we retrieved magazine media kits from publishers’ websites to obtain magazine readers’ demographic characteristics [44-63]. A media kit is a resource created by a publisher to help prospective ad buyers evaluate advertising opportunities [64].

This study used publicly available data and was deemed exempt from review by the University of Michigan Institutional Review Board.

## Results

This study examined 3 main themes and 8 subthemes related to women’s social roles and responsibilities, personal responsibility to improve health, and use of personal experience with PCOS to advocate for women’s health. This paper contains limited portions of publicly available magazine articles associated with the themes.

Table 1 lists the digital magazines included in the final sample, descriptions of the field served, total number of articles containing the keywords, and readership age and race or ethnicity. *Health*, *Parents*, *Women’s Health*, *Prevention*, *Shape*, and *Women’s Day* contained the most articles on PCOS.

**Table 1:** Publishers’ statements (January 30, 2014 to June 30, 2014 statement period) in magazines with articles on polycystic ovary syndrome (PCOS) and readership characteristics.^a^

Magazine	Field served	No. of articles on PCOS	Median age of readership (years)	Race/ethnicity of readership
*Better Homes and Gardens*	“*BETTER HOMES AND GARDENS* inspires women with infinite possibilities for creativity and self-expression. Each issue delivers smart, approachable editorial on design and individual style, decorating and gardening, food and entertaining, and personal and family well-being.”	5	51.02	NA^b^
*Cosmopolitan*	“*COSMOPOLITAN* focuses on personal growth, relationships and careers, with expanded reporting on fashion and beauty, health and fitness. Covered as well are celebrities and pop culture...and just about everything else women want to know about.”	3	34.7	NA
*Cosmopolitan en Español*^c^	“The assertive magazine for the independent Latin woman. Forward. Innovative. Successful. The Cosmo woman is all this and much more! *COSMOPOLITAN EN ESPAÑOL*, published as part of a joint venture with the Hearst Corporation, helps its readers to successfully balance their professional and personal lives. Editorial emphasis is on beauty, fashion and looking sensational.”	2	42.1	NA
*Essence*	“A lifestyle magazine for today’s African-American Woman.”	1	42.1	NA
*Family Circle*	“*FAMILY CIRCLE* speaks to moms of tweens and teens. *FAMILY CIRCLE* delivers advice for tough parenting challenges; provides suggestions for family activities; offers quick and healthy family recipes; and showcases projects to create a comfortable home. *FAMILY CIRCLE* features the latest health, diet, fitness and style news; and offers beauty and fashion tips.”	3	55	NA
*Fitness*	“A women’s magazine addressing fitness as a lifestyle.”	6	40	NA
*Glamour*	“Every issue of *GLAMOUR* includes news-making coverage of beauty, fashion, health and relationships as well as women’s issues, work, money and more.”	1	35.6	NA
*Good Housekeeping*	“Woman, Her Home and Her Family.”	4	55.5	White: 12,940 (83.5%); Black/African American: 1588 (10.2%); Spanish/Hispanic Origin: 992 (6.4%)
*Health*	“*HEALTH* is the magazine for women who have discovered a new kind of healthy living. It provides information and inspiration on all aspects of healthy living—from cutting-edge health ideas to food, fitness, beauty and relationships.”	30	49	NA
*MORE*	“*MORE* magazine is a fashion, beauty, trend and health guide for women of influence.”	4	54	NA
*O, The Oprah Magazine* “A Desirable Audience”	“*O, THE OPRAH MAGAZINE* covers 360 degrees of a woman’s life, from fashion and beauty, to relationships, food, home design, books, health and fitness, work and finance, technology, self-discovery and caring for others. The magazine encourages the reader to embrace her life, with the goal of becoming more of who she really is.”	4	50.6	African Americans: 4095 (34%)
*Parents*	“*PARENTS—*the magazine mothers with young children turn to for guidance and information needed to raise happy, healthy, well-adjusted children.”	24	34.9	NA
*Parenting* ^c,d^	“Reality-tested ideas and support for moms, by moms.”	11	NA	NA
*Prevention*	“Women’s magazine with interest in good health and personal fitness. Editorial focus on healthful foods, meal planning and preparation, nutrition, skin and body care, self-improvement and other topics contributing to a healthful lifestyle.”	16	57	NA
*Redbook* ^e^	“*REDBOOK* makes great style accessible for women aged 25 to 54. Editorial coverage includes beauty, fashion, home décor, fitness and nutrition, money management, relationships and personal growth.”	6	Women aged 35–49: 1722 (28.6%)	NA
*SELF*	“*SELF* is the magazine about the healthy well-being of the whole woman—body, mind and emotions. It encompasses everything that makes her unique, from her passions to her personal style.”	2	42	NA
*Shape*	“Young, educated, affluent women leading active lifestyles who use fitness, fashion, and beauty to be their best.”	15	39.6	NA
*Teen Vogue*	“A fashion and beauty magazine for teen girls. With a focus on fashion, coverage also includes entertainment and music, health and beauty, and inspiring profiles.”	1	27	NA
*Vanidades*	“A women’s beauty, fashion and lifestyle magazine in the U.S. Hispanic market. *VANIDADES* addresses the myriad interests of today’s woman. Beauty and fashion are at the forefront, featuring the most recent developments in cosmetology and the latest offerings from the most renowned fashion designers.”	1	39	Country/region of birth: Mexico: 40%; South America: 12%; United States: 11%; Central America: 8%; Cuba: 6%; Dominican Republic: 4%; Puerto Rico: 3% other: 16%
*Woman’s Day*	“Woman’s Service Field.”	10	55.5	NA
*Women’s Health*	“The *WOMEN’S HEALTH* brand was created for the woman who sees being healthy—physically and emotionally—as her edge. Our voice is how women speak to each other—with a tone and look that is uniquely *WOMEN’S HEALTH*.”	21	33	NA

^a^Readership age and race/ethnicity taken from magazine media kits. Readership demographics for digital (Web) issue audiences provided when available.

^b^NA: Not available.

^c^Media kit for *Cosmopolitan en Español* and *Parenting* unavailable.

^d^Publisher’s statement only available for the January 30, 2013 to June 30, 2013 statement period.

^e^Median age unavailable.

### Women’s Social Roles and Responsibilities

PCOS was depicted as a barrier to childbearing, starting a family, and effective breastfeeding. In turn, these issues hindered women’s roles as wives and mothers. A total of 28 instances of this theme emerged from 9 magazines.

#### Childbearing and Role as a Wife

Childbearing was depicted as a wife’s responsibility. Women’s inability to have children lessened women’s worth as wives. *Essence* published the following in a piece titled *Steve Harvey Morning Show’s Daily Strawberry Letter*, a radio show in which listeners submit letters online describing personal issues and problems, in hopes that hosts Steve Harvey and Shirley Strawberry will give them advice:

Today’s topic: Less Than A Wife

Hello Steve, Shirley, and Morning Crew. I am a 39 yr old woman with a wonderful husband. He is a God fearing man, that is an excellent provider, and the best husband that any woman could ask for. He is the most unselfish person that I have ever met. We desperately want to start a family but this past summer I was diagnosed with diabetes and ended up in ICU [intensive care unit] with a blood sugar of 980…... I also have PCOS (Polysystic Ovary Snydrome [sic]) which is already an issue and now this. I feel like less than a wife because I can not give my husband the one thing that he wants the most. [65]

Use of “wonderful” suggests the writer holds her husband in high regard. Her description of him as “an excellent provider” and as “the best husband that any woman could ask for” also suggest she perceives him as meeting his role and responsibilities as a husband. The writer’s tone becomes somber as she blames her poor health for preventing pregnancy and PCOS as an additional barrier to starting the family she and her husband “desperately want.”

#### Childbearing and Starting a Family

Women’s primary motivation to lose weight and manage PCOS symptoms was to increase their likelihood of pregnancy and form a family. The following excerpt published in *Fitness* featured Olivia Ward, season 11 winner of *The Biggest Loser*, a reality weight-loss show:

Olivia, who suffers from polycystic ovary syndrome, a condition that causes ovarian cysts and often disrupts the menstrual cycle, went on the show because doctors told her she needed to lose weight if she hoped to improve her chances of getting pregnant. She’s now looking forward to starting a family with Ben. [66]

The excerpt indicates that Olivia was unable to have children as an obese woman with PCOS. PCOS is depicted as a barrier to starting a family with her husband.

#### Breastfeeding

In 2 instances, PCOS was depicted as a barrier to effective breastfeeding. In the following excerpt, *Parents* magazine responds to the following reader’s question: “Is breastfeeding when overweight difficult?”:

Most plus-size women can breastfeed just as well as other women. However, if you’re overweight because of a hormone or endocrine problem, like polycystic ovary syndrome (PCOS) or thyroid disease, you may have issues with your milk supply, since these conditions can also affect production of the hormones that trigger your body to make milk. But having PCOS, thyroid disease, or other hormone-related illnesses doesn’t necessarily mean you won’t be able to breastfeed. You may just have to supplement more often with formula to keep your baby fully nourished. If you’re worried, it’s a good idea to talk to a lactation consultant to help you and your baby get the hang of nursing if you run into problems. [67]

The reader is concerned that being overweight could affect breastfeeding. The piece depicts PCOS as a potential barrier to nursing if it limits milk supply, a barrier that can affect the mother’s caregiving and the child’s nutrition.

### Personal Responsibility for One’s Health

Women were depicted as responsible for improving their own health through diet, exercise, hard work, and seeking good-quality health care. A total of 109 instances of this theme emerged from 17 magazines.

#### Lifestyle Changes

Magazine articles largely placed personal responsibility on women to improve their health with lifestyle changes. Women’s stories illustrated ways in which women changed their diet and exercise through hard work and dedication. The following piece, titled *12 crazy-inspiring photos and details of weight loss success stories*, appeared in *Women’s Health*:

The Lifestyle: Courtney dove into daily workouts. She jumped rope, punched bags, and learned how to fight. She fueled up with veggies and lean protein, and gave up ice cream after learning to satisfy her sweet tooth with fruits like pineapple.

The Reward: Boxing also gave Courtney a major self-esteem boost. “I used to be afraid of talking in front of people,” she says, “but now I’m confident.” And although her PCOS is permanent, she feels healthy and has tons of energy. “I know I can face anything,” she says. “Just like in the ring, you can always go one more round. You just have to dig for it.” [68]

Courtney’s boxing is a metaphor for her “fight” against PCOS, a chronic condition Courtney is confident she can “face” or manage each “round.” Courtney’s hard work and dedication in adopting a healthy lifestyle was akin to training for a boxing tournament, in which she learned to fight an opponent. In this case, poor diet and inactivity were the opponents or barriers to weight loss. “You just have to dig for it” suggests that Courtney views one’s ability to change as a result of hard work.

#### Good-Quality Health Care

Articles also largely placed personal responsibility on women to seek good-quality health care. The following piece, titled *Could you have PCOS?*, appeared in *SELF*:

The key is to see a health care provider with knowledge of the condition (ask whether the doctor frequently sees patients with PCOS). You can also request a referral to an endocrinologist. And if you do have PCOS, remember: The condition can be controlled, but it takes discipline. [69]

The piece places responsibility on women to find a knowledgeable health care provider and request referrals to specialists. Additionally, the piece reminds women that PCOS can be controlled with “discipline,” suggesting that self-control is a primary factor in managing health.

### Personal Experience as a Catalyst for Advocacy

Women were portrayed as using their personal experience with PCOS to promote women’s health by sharing knowledge and expertise, promoting acceptance of one’s body, and creating dialogue about miscarriage and infertility. A total of 8 instances of this theme emerged from 5 magazines.

#### Sharing Knowledge and Experience

Personal stories depicted women’s knowledge of and experience with PCOS symptoms as valuable. Women expressed a desire to share their knowledge and experience with other women.

The following is an excerpt of a YouTube video embedded in a *Redbook* article titled *“I’m giving infertility a voice.” Carla opens up about her struggle with polycystic ovarian syndrome*. Carla begins by telling viewers “I wish I had known that infertility isn’t something to be ashamed of.” She goes on to say she is 28 years old and has been diagnosed with PCOS and factor V Leiden, a condition that can increase one’s risk of developing abnormal blood clots [70]. Then, Carla reflects on her experience with PCOS-related infertility:

Infertility can be really lonely. Besides the shame you might feel, there’s a lot of misconception and misinformation floating around. I made it my mission to combat that and give infertility a voice by sharing my journey and educating those around me. I’m really proud of myself that I can look back on my journey to motherhood and know that not only did I become more resilient as an individual, and not only did it strengthen my marriage, but I was able to give hope to other people by educating them about infertility. I wish I hadn’t been so ashamed about my diagnosis, but I’m glad I eventually realized, it’s not my fault. [71]

Carla characterizes infertility as a lonely and shameful experience. The video suggests that Carla experienced shame as a result of her inability to conceive and fulfill her gender roles as a woman, mother, and wife. The video also suggests that Carla’s distressing experiences with infertility created a sense of duty and responsibility to “give hope” to women struggling with infertility. The excerpt concludes with Carla expressing happiness about realizing she was not to blame for her diagnosis of infertility, a rare contrast to the discourse on personal responsibility for one’s health.

#### Promoting Acceptance

Encouraging women to accept their bodies was another way in which articles portrayed women as advocates for women’s health. For instance, *Health* published a piece featuring Whitney Thore, the star of the reality TV show *My Big Fat Fabulous Life*, and her efforts to embrace her body [72]. Whitney gained notoriety when she posted a YouTube video titled *A Fat Girl Dancing*, in which she danced to a popular pop song [73]. According to *Health*:

Her videos became so popular that the now 30-year-old Thore launched the #NoBodyShameCampaign, a movement that encourages self-love and acceptance no matter your size or gender.

Even Thore admits her weight gain wasn’t due solely to PCOS. “Now I’ve gained 200 pounds so certainly I take personal responsibility. That’s not all because of a medical condition,” Thore told the Today Show last year. “But definitely I felt so ashamed once I started gaining weight that I was too embarrassed to even go to a doctor.” If you think you’ve put on too much weight, it’s important to take the time to get it checked out. [74]

Thore used her knowledge of and experience with PCOS, as well as her popularity, to launch a campaign that promotes love and acceptance of one’s body. Thore’s campaign challenges social norms of what ideal bodies should look like. Use of “personal responsibility" suggests Thore believes personal choices, perhaps lack of self-discipline and control, contributed to her weight gain.

#### Creating Dialogue

Women’s magazines also featured personal stories of celebrities who, unlike Thore, were thin and had no visible signs of PCOS. This included a piece in *Parents* magazine titled *Jaime King opens up: miscarriage, IVF—and then natural conception* featuring actress and model Jaime King:

The 35-year-old actress speaks openly about the traumatic process in this week’s People magazine, revealing that she had five miscarriages, endured five rounds of IVF [in vitro fertilization], and 26 rounds of IUI [intrauterine insemination]. She says that a diagnosis of endometriosis and polycystic ovary syndrome were behind her difficulties.

So with a healthy and happy 1-year-old baby in her life, and that suffering behind her, why is she talking about her miscarriage experience now? Because she says she wants to open up lines of dialogue about a topic that can be stubbornly closed for suffering hopeful moms-to-be out there. “I was hiding what I was going through for so long, and I hear about so many women going through what I went through,” she told the magazine. “If I’m open about it, hopefully it won’t be so taboo to talk about it.” [75]

King publicly discloses her PCOS diagnosis as an underlying cause of her infertility. “Traumatic” suggests that King’s experiences with miscarriage and forms of assisted reproductive technology such as in vitro fertilization and intrauterine insemination negatively affected her physical and emotional well-being. King’s motivation for publicly sharing her experience is to “open up lines of dialogue” about infertility and facilitate dialogue about a topic socially unacceptable or forbidden to discuss publicly.

## Discussion

To our knowledge, this study is the first to explore PCOS content in digital teen and women’s magazines. The presence of PCOS content in digital magazines in this study counters previous findings that women’s magazines tend to feature health content that is “old news,” that is, information the public is generally familiar with [33]. PCOS is far from being old news to health care providers, patients, and the general public. Indeed, PCOS is a topic that within the last few years medical experts have referred to as a “hot topic” and “new frontier” in the area of female fertility [76].

This study found that digital magazines depicted PCOS as a barrier to having children that hindered women’s roles as wives and mothers. These findings are consistent with Williams and colleagues’ findings that women perceive PCOS as interrupting their plans to start a family and the timing of starting a family [23,77]. The portrayal of PCOS as a possible barrier to breastfeeding in women’s magazines supports nascent research that suggests that women living with PCOS experience breastfeeding challenges. These challenges include low breast milk supply, limited ability to bond with their children through breastfeeding, and perceptions that breastfeeding can increase their daughters’ risk for developing PCOS [78].

Portrayals of adolescents with PCOS were largely absent from magazine articles included in this study. This may be because teen magazines such as *Teen Vogue* primarily focus on clothing, shopping, and cosmetics [79]. In addition, the underrepresentation of adolescents in magazine articles may be associated with the limited research on PCOS in adolescence and its impact on health and mental health across the life course. Lack of research in these areas is one of the reasons why the NIH Evidence-based Methodology Workshop on Polycystic Ovary Syndrome has recommended conducting basic and translational research on PCOS in adolescence and the long-term consequences of PCOS diagnosed in adolescence [5]. This lack of research, combined with the challenges of diagnosing PCOS in adolescence [20], and the confusion surrounding the name “PCOS” [80,81] can affect the extent to which research trickles down to media such as magazines.

The absence of discourse on race and ethnicity, and the absence of PCOS content in magazines directed toward Latinas and African American women, suggests that portrayals of women with PCOS largely focus on the white body. This is cause for concern because Latinas and African American women are particularly at risk for PCOS due to high rates of obesity and metabolic problems (eg, insulin resistance, hypertension) [82] among these groups, and screening rates for metabolic problems among racial and ethnic minority women with PCOS tend to be low [83]. Interestingly, this study did not find articles depicting stories of African American women with PCOS in *O, The Oprah Magazine*, a magazine whose media kit claims to reach more African American readers than *Glamour*, *Redbook*, *SELF*, and *MORE* [54]. In addition, this study did not find articles depicting Latina or Spanish-speaking women’s experiences with PCOS in either *Cosmopolitan en Español* or *Vanidades*, magazines published in Spanish and directed toward Latinas. The findings suggest that while discussion on race, ethnicity, and PCOS is not absent from the medical literature, it is absent in this type of popular media.

Magazine articles included in this study placed considerable demands on women to take responsibility for their own health. The discourse on personal responsibility has been observed in other research with women’s magazines. For instance, a review of readers’ letters to women’s health magazines found that writers tended to attribute their successes and failures in managing their health to individual behavior [30]. In addition, a content analysis of over 400 editorials with content on diet, overweight, and obesity published between 1984 and 2004 in mainstream and African American women’s magazines found that these editorials primarily provided readers with strategies to change individual behavior such as reducing fast food intake and eating smaller portions [84]. Personal responsibility is certainly important in managing one’s health. Indeed, women with PCOS report that diet and exercise are critical in controlling their weight and other PCOS symptoms [77]. However, it is important to keep in mind that personal responsibility discourse that blames individuals for poor lifestyle choices and poor health can perpetuate stigma and discrimination toward obese individuals and hinder efforts that address environmental and structural barriers to health [85,86].

The discourse on economic and environmental barriers to health was largely absent from teen and women’s magazines. These barriers include high levels of food toxins and environmental exposure to industrial products (eg, bisphenol A in plastics) that appear to play a role in the development of PCOS [87], although further research in this area is needed. Additional barriers to health include lack of insurance coverage for infertility-based treatments and assisted reproductive technologies [88], neighborhood crime that limits one’s ability to exercise in the community, and limited access to fresh fruits and vegetables [84].

Magazine articles depicted women as using their personal experiences with PCOS to be advocates for women’s health. These women attached their names, voices, faces, bodies, emotions, and personalities to PCOS by making their most personal, private experiences with PCOS (eg, infertility, obesity, and poor body image) public. These women appear to have engaged in meaning making, a process in which individuals try to make sense of or comprehend a stressful event and search for the value or significance of the event in their lives. Meaning making can result in acceptance of the event, personal growth, enhanced coping skills, a greater appreciation for life, and a change in identity [89]. In their journey with PCOS, women gained power and control over their body and their lives, and realized that the knowledge and experience they gained during their journey was valuable and should be shared with others to educate them about complex health issues, give others hope, and facilitate dialogue about taboo topics.

### Study Limitations

A limitation of this study was the 1-month search period that did not lend itself to the study of potential changes in PCOS content over time. Articles on PCOS were included in the study if they were available online during the 1-month search period. As a result, the articles included in this study may not be representative of PCOS content published in these media over a longer period of time. Despite this limitation, the 1-month search period was effective in demonstrating that, indeed, women’s digital magazines publish content on PCOS.

This study also found that, in some cases, articles were published online months and even years preceding the search period. This illustrates how commercial publications such as magazines that once had short shelf lives as hard copies can continue to be accessible online for lengthy periods of time [90].

A second limitation was the method used to search for articles using the keywords “PCOS” and “polycystic ovary syndrome.” We used only these keywords in this study. This may have excluded articles containing other names for PCOS such as “polycystic ovarian syndrome.” In addition, the search method may not have yielded all articles containing the keywords. For instance, an article titled *7 celebrities who manage life with chronic pain* published in November 2011 in *Prevention* magazine containing the keywords was not returned by the magazine website’s search engine using this search method. As a result, we did not include this article in this analysis. This indicates the search bars in digital magazines may not have been sensitive enough to find all articles containing the keywords. Future research should include a more rigorous search method.

Future research should also examine how readers respond to PCOS content in digital teen and women’s magazines. This includes ways in which readers respond to women’s personal stories and manage their health, or not, as a result of exposure to this content. Research in this area is especially important because the number of Internet users of online content to make health and health care decisions is rising [91].

### Conclusion

This study highlights how digital teen and women’s magazines can be used to understand health communication about PCOS. Research on popular media content is not readily available in the literature on PCOS, as much of the information on women with the condition comes from medical sources. This study found a lack of representation of adolescents and racial and ethnic minority women with PCOS in magazine articles. In addition, this study found social values and beliefs about women with PCOS embedded in the articles. The findings can inform health education programs that teach women to be critical consumers of PCOS-related content in digital women’s magazines. Critical consumers have the skills to recognize how content is socially constructed and make informed decisions about their health through reflection, questioning, and analysis of media [92]. Future research on PCOS content in digital teen and women’s magazines can help researchers, patients, and consumer groups engage with the media to increase public awareness of PCOS.

## Abbreviations

AAM: Alliance for Audited Media
NIH: National Institutes of Health
PCOS: polycystic ovary syndrome
